# Transition from military to civilian: Identity, social connectedness, and veteran wellbeing

**DOI:** 10.1371/journal.pone.0261634

**Published:** 2021-12-22

**Authors:** Mal Flack, Leah Kite

**Affiliations:** College of Health and Human Sciences, Charles Darwin University, Darwin, Northern Territory, Australia; Julius-Maximilians-Universität Würzburg, GERMANY

## Abstract

Military identity and a sense of social connectedness may help explain differences in contemporary veteran wellbeing following transition from military to civilian life. However, it is unclear how these constructs interrelate. The current study quantitatively explored the role of social connectedness in the relationship between military identity and subjective wellbeing among contemporary ex-serving Australian Defence Force veterans. To facilitate analyses, data from 358 veterans were used to first explore the suitability of the factor structure of the Warrior Identity Scale. Subsequently, the potential moderating and mediating effects of social connectedness in the relationship between military identity and wellbeing were explored via path analysis. Confirmatory factor analysis of the Warrior Identity Scale revealed support for the multidimensional construct of military identity, and a revised six-factor measurement model was found suitable for further path analysis. Consistent with past research, social connectedness positively related to quality of life and negatively related to psychological distress. There was no support for a moderation effect of social connectedness. However, results indicated military identity indirectly influenced wellbeing and distress via differential relationships with social connectedness. Specifically, private and public regard for the military and not feeling like an outsider positively related to social connectedness. In contrast, interdependence with other veterans, viewing the military as family, and the centrality of military identity negatively related to social connectedness. The results suggest nurturing the protective aspects of military identity and addressing inhibitory aspects of military identity may support a sense of social connectedness and wellbeing among ex-serving veterans.

## Introduction

Research indicates a significant proportion of contemporary veterans experience challenges following transition from military to civilian life that diffuse across multiple areas of functioning to impair wellbeing [1–11]. Understanding risk and protective factors for wellbeing among contemporary ex-serving Australian Defence Force veterans is important because research indicates this cohort are at a greater risk of psychological distress and lower wellbeing compared to serving veterans and the Australian community [1–3, 6, 11–13]. Although a range of individual differences contribute to veteran functioning following transition, these studies indicate effective identity and social adjustment are fundamental to wellbeing. Furthermore, quantitative research demonstrates aspects of an ensuing military identity and a sense of social connectedness influences wellbeing among contemporary ex-serving veterans [14–19]. However, few studies have quantitatively explored how these two constructs interrelate to influence contemporary veteran wellbeing following transition to civilian [5, 16, 17].

### Social identity approach

The current study explored the role of social connectedness in the relationship between military identity and wellbeing within a social identity approach framework [20–22]. The social identity approach draws on social identity theory [23] and self-categorisation theory [24] to explain the formation and dynamic nature of identity in a real or perceived social context. Integral to the social identity approach is the social identity model of adjustment to identity change [21, 22]. According to the social identity model of adjustment to identity change, the impact of transition stress is influenced by the subjective evaluation of the capacity to activate or adopt social identities congruent with the new environment. Research centred on the social identity model of adjustment to identity change reinforces the protective nature of malleable social identities and identification with various social groups, because these factors assist to retain social connectedness and social resources through stressful life transitions in support of wellbeing [25–29].

Similarly, military transition theory, as defined by Castro and Kintzle [30], reinforces the importance of identity malleability and establishing a sense of belonging to the transitioned environment. Framed within the social identity approach, military transition theory posits these factors are vital to effectively navigate identity and social change in support of wellbeing across all phases of transition from military to civilian life. Furthermore, Thompson et al. [10] maintain the social identity model of adjustment to identity change complements military transition theory and may be an important framework for conceptualizing and investigating factors that impact effective identity and social adjustment relating to the transition from military to civilian life. Collectively, these approaches indicate a need to examine how aspects of an ensuing military identity might interrelate with a sense of social connectedness to the broader social world to influence the wellbeing of contemporary ex-serving veterans.

### Social connectedness

A sense of social connectedness is intrinsically linked to the social identity approach and social identity model of adjustment to identity change [21, 22]. As defined by Lee and colleagues [31–33], social connectedness develops according to a collective of subjective interpersonal social experiences that guide patterns of responding to the social world. From this perspective, social connectedness reflects the individual’s perception of the social world, and the degree to which they feel interpersonally connected to and belong to the social world. Although personality traits such as extraversion are thought to contribute to the development of social connectedness, Lee, Dean, and Jung [34] maintain extraversion and social connectedness are different constructs. Furthermore, social connectedness is considered a global construct that accounts for proxy elements such as loneliness, social integration, and social support resources [31–33, 35–37]. Therefore, exploring social connectedness from a broader frame of reference may be vital to examining interpersonal factors contributing to belonging and disconnection from society.

Among ex-serving veteran populations, higher social connectedness has been associated with lower PTSD symptom severity [38] and lower suicidality [6, 19]. Additionally, the relationship between moral injury perpetrated by others and suicidality among ex-serving US combat veterans has been shown to weaken as a function of higher social connectedness [15]. More broadly, higher social connectedness has been associated with adaptive interpersonal functioning, actively seeking to connect and belong to the social world, and happiness [31, 33, 35, 36, 39–41]. Furthermore, an extensive body of evidence indicates social connectedness is not only important to wellbeing, but to survival [35, 36]. Interestingly, the perceived or real capacity to integrate with social networks is a stronger predictor of wellbeing than received social support [36]. Lee and colleagues [31–33] suggest low social connectedness may explain why an individual with access to social resources may still feel isolated and disconnected from the world. As such, social connectedness is argued to be central to understanding the relationship between an ensuing military identity and veteran wellbeing following transition from military to civilian life.

### Literature review

It is widely accepted that a military identity is a social identity, formed as the civilian identity becomes less salient through military enculturation and is integrated into the self-concept [10, 42–45]. Shared military experiences, social bonds and trust, and the importance of the military to the veteran operate to reinforce the salience of the military identity in the self-concept [42, 43, 46]. Additional reinforcers include length of service and number of deployments [18], and few opportunities to disconnect from military life [47, 48]. The salient military identity is protective for serving veterans because it is congruent with the requirements of military service [47, 49–52]. However, military enculturation and a dominance of the military in the veteran’s life may contribute to a problematic blending of the personal and social identities, further entrenching military identity in the self-concept impairing effective identity and social adjustment [5, 30, 42, 45–47, 53–58].

Similar to social identity model of adjustment to identity change research, interpretive phenomenological analysis has shed light on the inhibiting effects of a salient military identity and disconnectedness from civilian life on the capacity of ex-serving veterans to effectively adjust to identity and social change in support of wellbeing [53, 57]. For example, McCormack and Ell [57] found identity change related to themes of loss, social isolation, and poor wellbeing, as well as psychosocial transition challenges among ex-serving veterans. Thematic analysis revealed a salient “Warrior” (p. 246) identity following transition may further function to rationalise self-doubt associated with military related moral injury. This strategy in conjunction with an unwillingness to relinquish the dominance of the military identity reflected social disconnectedness through social withdrawal or anti-social behaviour and a reinforcement of the military-civilian divide. Subsequently, disconnectedness from both the former and new social environments inhibited efforts to re-connect psychosocially in support of personal growth. Similarly, Binks and Cambridge [53] found a persistent military identity among ex-serving UK veterans who engaged in strong military social bonds and viewed their military role as fundamental to identity while serving experienced greater psychosocial transition adjustment challenges. Consistent with previous research, identity disruption was associated with the loss of the military in the veteran’s life and a perceived disconnectedness or rejection from society. Semi-structured interviews by Smith and True [45] revealed similar trends among ex-serving US veterans. Trust and strong bonds with other veterans formed during service and a void of non-military social networks reinforced the salient military identity and a sense of disconnection from family and society, impairing wellbeing.

Exploring how a sense of social connectedness may interrelate with military identity may shed light on factors that differentiate veterans who express the desire to push through identity challenges and veterans who withdraw socially or demonstrate resistance to change. For example, Dabovich, Eliott, and McFarlane [59] identified the capacity to integrate military and civilian value systems to cope with identity change was protective among male Australian Army veterans. Veterans were undergoing combat-related rehabilitation and were either transitioning out of the military or preparing to return to duty. Semi-structured interviews revealed themes of pushing boundaries, personal agency, and personal desire to evolve through change supported effective transition among the veterans. Demers [56, 60] also reported themes of a strong desire to adopt blended military-civilian identities integrative of both value systems among ex-serving US veterans through particularly stressful identity conflicts. McCormack and Ell [57] highlighted similar themes of an underlying desire to move forward through stressful identity change and re-establish the self beyond the military. Furthermore, Brinn and Auerbach [61] found social networks assisted US veterans to find meaning in their military service and strike an effective balance between their military and civilian identities in support of wellbeing through transition. It is conceivable individual differences in social connectedness may have contributed to the findings of these studies.

Large scale qualitative and mixed-methods research have reinforced the protective nature of malleable social identities and balanced social networks following transition from military to civilian life. For example, Brewer and Herron [48] found a centrality of military identity at the expense of alternate social or personal identities related to greater transition challenges and poor wellbeing among ex-serving UK veterans. In contrast, veterans who maintained a balanced approach to military and civilian life and viewed the military from a vocation lens fared better. Remaining connected to the civilian world during service appeared to facilitate the capacity to separate the military self from the civilian self, as evidenced by better adjustment to civilian life among reserve veterans compared to regular veterans.

Other large-scale US transition studies have also found a salient military identity to be entrenched in both personal and social identities, further inhibiting the formation of new identities and community integration among ex-serving veterans [5, 62, 63]. Furthermore, this blending of the personal and social identities presents a considerable risk to wellbeing among this cohort because low social connectedness is a significant risk factor for suicide among veteran populations [6, 18, 19, 30]. An extensive body of evidence indicates a broader cohort of ex-serving veterans experience deficits in a range of aspects associated with social connectedness that differ markedly from serving veterans and non-military populations, due to multiple transition losses including the loss of naturally occurring social networks that once served as a protective factor [37, 44, 64, 65]. Additionally, avoidant behaviour associated with low social connectedness coupled with the loss and separation is likely to exacerbate transition stress and poor wellbeing [66]. As such, ubiquitous access to naturally occurring social networks during military service may mask deeper social connectedness issues that might only be apparent through transition [36].

In a large-scale quantitative study, Adams et al. [14] found some protective effects of a salient military identity among ex-serving US contemporary and Vietnam veterans. Adams et al. [14] employed a 3-item scale to assess the subjective importance or centrality of the military identity. High centrality of military identity was associated with lower suicidality, uptake of Veteran Affairs’ services, and higher perceived levels of unit support and morale during service. However, centrality of military identity was not associated with measures of subjective wellbeing, depression, or PTSD. Although this study highlighted the potential for military identity to influence help-seeking in support of wellbeing, Adams et al. acknowledged it may also be helpful to investigate how other aspects of identity relate to wellbeing.

In contrast to Adams et al. [14], Kreminski et al. [16] found a positive relationship between Soldier identity and depression. Additionally, a protective effect of the perceived connectedness to the former military network was evidenced by a negative relationship with depression. The regression model indicated Soldier identity explained 5.8% of the variance in depression, and perceived connectedness a further 11% of the variance. The disparate findings of the Kreminski et al. [16] and Adams et al. [14] studies support the notion that different aspects of military identity may have dissimilar relationships with social connectedness and wellbeing among veteran populations.

Although scarce, scale development research viewing military identity as a multidimensional construct has expanded on earlier findings by investigating aspects of military identity that may support or inhibit veteran wellbeing [17, 18, 50, 51, 67]. Johansen et al. [67] developed the culturally specific multidimensional identity scale to assess aspects of military identity central to national and military culture among serving Norwegian Armed Forces. Importantly, research supported the multidimensional nature of military identity, and indicated elements differentially influenced veteran functioning. For instance, the identity subscales have demonstrated differential predictive validity across a range of important military service factors among serving members of the Norwegian Armed Forces [50, 67] and among serving and ex-serving members of the UK Armed Forces [68].

The Warrior Identity Scale (WIS) [17, 18] is another multidimensional instrument that was developed to assess military identity in an American context. The WIS was guided by measures of ethnic and vocational collective identity, which was modified to suit a military population. Lancaster and Hart [17] conducted a pilot study based on a subset of the original 66 WIS items to explore relationships between psychological functioning and social support among ex-serving US veterans. Psychological functioning and uptake of social support following deployment were positively related to high private and public regard for the military. In contrast, depression, PTSD severity, and negative affect were positively associated with interconnectedness with other veterans and viewing the military as family.

Lancaster et al. [18] further developed the WIS using a revised subset of items to explore the factor structure and relationships between aspects of military identity and psychosocial wellbeing among ex-serving US veterans. Results were consistent with the pilot study. High private and public regard, and not feeling like an outsider were associated with less transition adjustment challenges, lower depression and PTSD symptom severity, less somatic symptoms, lower suicidality, and greater life satisfaction. In contrast, viewing the military as family and identity exploration were associated with adjustment difficulties, greater depression and PTSD symptom severity, greater somatic symptoms, higher suicidality risk, and lower life satisfaction. Although the WIS demonstrates potential utility in an Australian context, the scale had not previously been examined cross-culturally and was still under development.

Further exploratory analysis based on large scale US transition study data provided support to the notion that diverse aspects of military identity may have different associations with social connectedness and suicidality [19]. The analysis revealed low private and public regard for the military and greater interdependence with other veterans was associated with low social connectedness. Whereas low social connectedness, low private regard, and high interdependence significantly predicted risk of suicide in the sample. Furthermore, participants were seven times more likely to report clinically significant suicidality if they reported low social connectedness. A subsequent study found support for a mediating effect of social connectedness in the relationship between two military specific factors and PTSD symptoms among ex-serving US veterans [38]. That is, both non-honourable discharge status and combat experiences negatively predicted social connectedness, which, in turn, negatively predicted PTSD symptoms. Other research has shown social connectedness may moderate the relationship between service factors and wellbeing. For example, the relationship between moral injury perpetrated by others and suicidality has been found to weaken as a function of higher social connectedness (15).

Framed within military transition theory [30] and the social identity model of adjustment to identity change [21, 22], two important themes emerged from past research. First, aspects of military identity can persist after discharge that may support or inhibit effective social adjustment and wellbeing. Second, an individual’s sense of social connectedness to the broader social world relates to wellbeing; however, may also interrelate with aspects of military identity to influence the perceived capacity or desire to identify with and connect to the transitioned environment. However, uncertainty exists as to whether social connectedness moderates or mediates the relationship between military identity and wellbeing. Furthermore, quantitative investigation of the interrelationships between the multidimensional construct of military identity and a sense of social connectedness to the broader social world are scarce [19]. As such, the purpose of the current study was to expand on past research by first examining the suitability of the factor structure of the WIS in an Australian Defence Force context, followed by exploration of potential moderation and mediation effects of social connectedness in the relationship between military identity and subjective wellbeing via path analysis.

## Materials and methods

### Ethics statement

The current study was conducted in accordance with the ethics approval granted by the Departments of Defence and Veterans’ Affairs Human Research Ethics Committee (DDVA HREC) (Protocol 108–19). Prior to commencing the survey, participants were asked to read Participant Information Sheet and indicate they had and understood the information and agreed to proceed by making the appropriate selection on the online survey.

### Participants

Participants were contemporary ex-serving Australian Defence Force veterans who had rendered full-time service and discharged from the Australian Defence Force between 2000 and 2019. A total of 358 veterans completed the anonymous online survey. The majority of participants (65.8%) reported discharging between 2011 and 2019, with an average time in service of 14 years (*SD* = 9). Participants identified as Indigenous Australians (1.7%), of Western European decent (88.5%), or other ethnic backgrounds (9.8%) (aggregated). Ages ranged from 20 to 66 years or older with a mean age of 42 years (*SD* = 9). Participants included 111 females and 246 males (one participant did not disclose gender). The sample included ex-serving veterans who served in the Australian Army (69.8%), Royal Australian Navy (18.7%), Royal Australian Air Force (8.9%), and 2.5% reported service in more than one branch of the Australian Defence Force. A comprehensive sample profile is detailed in Table 1.

**Table 1:** Sample profile (*N* = 358).

Element	*n*	%	*M*	*SD*
Age	-	-	42	9
Female	111	31	-	-
Male	246	68.7	-	-
Did not disclose gender	1	.3	-	-
Royal Australian Navy	67	18.7	-	-
Australian Army	250	69.8	-	-
Royal Australian Air Force	32	8.9	-	-
Regular military service in more than one branch	9	2.5	-	-
Discharged more than once	41	11.5	-	-
Length of military service	-	-	14	9
Commissioned Officer	51	14.2	-	-
Warrant Officer	43	12	-	-
Senior Non-Commissioned Officer	58	16.2	-	-
Non-Commissioned Officer	94	26.3	-	-
Other Rank	112	31.3	-	-
Discharged voluntary	201	56.1	-	-
Discharged medical	124	34.6	-	-
Discharged involuntary	9	2.5	-	-
Reached compulsory retirement age or self-funded retirement	6	1.7	-	-
Other reason for discharge	18	5	-	-
Did not deploy	67	18.7	-	-
Non warlike/active service (e.g. peacekeeping, UN assistance missions)	136	38	-	-
Warlike/active service	223	62.3	-	-
Exercise outside of Australia	135	37.7	-	-
Border protection	83	23.2	-	-
Humanitarian assistance/disaster relief	70	19.6	-	-
Defence aid to the civilian community	96	26.8	-	-
Other deployment type (e.g. exchange)	15	4.2	-	-
Highest education level completed “university degree”	96	27	-	-
Highest education level completed “diploma” or “certificates”	210	59	-	-
Highest education level completed “secondary” or “primary”	52	14	-	-
Relationship status either “partner”, “de facto”, or “married”	289	80.7	-	-
Employed within six months of discharge (as applicable)	213	59.5	-	-
Employed full-time	176	49.2	-	-
Employed part-time	27	7.5	-	-
Student	18	5.0	-	-
Medical pension and not working	52	14.5	-	-
Retired	22	6.1	-	-
Unemployed	16	4.5	-	-
NT	35	9.8	-	-
QLD	100	27.9	-	-
NSW	76	21.2	-	-
ACT	22	6.1	-	-
VIC	60	16.8	-	-
TAS	6	1.7	-	-
WA	31	8.7	-	-
Location other than Australia	7	2.0	-	-
Family depends on financially “yes” or “partially”	243	67.8	-	-
Self-report chronic illness or injury “yes”	220	61.5	-	-
Social interaction with serving or ex-serving ADF members (veterans) “never”	16	4.5	-	-
Social interaction with veterans “rarely” or “yearly”	125	35.0	-	-
Social interaction with veterans “monthly” or more	218	60.6	-	-
Satisfied with transition experience	136	38.0	-	-
Dissatisfied with transition experience	222	62.0	-	-
Felt they had not transitioned to civilian life	73	20.4	-	-
Felt they had transitioned to civilian life	137	38.3	-	-
Felt they had partially transitioned to civilian life	148	41.3	-	-
Rated overall quality of life as “good” or higher	214	59.8	-	-
Rated overall satisfaction with health as “satisfied” or higher	117	32.7	-	-

### Measures

#### Warrior identity scale

The WIS was developed as a multidimensional measure of military identity [17, 18]. As the WIS was still under development, a subset of 31 items from the initial pool of 66 items were provided for the current study (March 29, 2019, email from Lancaster, S.L., Assoc Professor and Chair, Department of Psychology, Bethel University MN: steven-lancaster@bethel.edu). Items are rated on a 4-point response scale from 1 = disagree strongly, 2 = disagree, 3 = agree, 4 = agree strongly. The 31 items reflected seven domains of military identity referred to as private, public, connect, interdependence, family, centrality, and skills. Private regard for the military contained items such as “I am proud to have served in the military”. Public regard for the military contained items such as “society views veterans as an asset”. Connection with other military members was designed to reflect a lack of feeling like an outsider and contained items such as “I never felt emotionally connected to my military unit”. Interdependence with other military members contained items such as “Only other veterans can truly understand me”. Viewing the military as family contained items such as “By leaving the military I lost a family”. Centrality of military identity included items such as “In general, being a veteran is an important part of my self-image”. Skills learnt in the military included items such as “I appreciate the skills I learned in the military”. Scores are scaled in a positive direction, with higher scores indicating a higher degree on the subscale. Following confirmatory factor analysis, subscales were computed. Cronbach’s alphas are detailed in the results.

#### Social connectedness scale–revised

The SCS-R [31] is a 20-item self-report scale designed to measure the degree to which individuals perceive they are interpersonally connected and belong to the social world. Items are rated on a 6-point response scale ranging from 1 = strongly disagree to 6 = strongly agree. Scores are summed to provide a total score ranging from 20–120. The total score is scaled in a positive direction, with higher scores indicating higher social connectedness. Cronbach’s alpha in the current study was .96.

#### Kessler psychological distress scale (K10)

The K10 is a 10-item scale designed to measure non-specific psychological distress over a previous 30-day period [69]. Items are rated on a 5-point scale ranging from 1 = none of the time to 5 = all of the time. The total score is scaled in a positive direction, with higher scores indicating higher psychological distress. Scores are summed to provide a total score ranging from 10–50. Cronbach’s alpha in the current study was .95.

#### WHO quality of life Bref (WHOQOL-Bref)

The WHOQOL-Bref is a short 26-item scale adapted from the WHOQOL-100 designed to measure subjective quality of life across four domain profiles of physical health, psychological, social relationships, and environment [70, 71]. Two items are separately scored to provide an overall indication of an individual’s perception of health and quality of life (see Table 1). Rating scales differ among the items. Domain scores are scaled in a positive direction, with higher scores indicating higher perceived quality of life. Sample mean scores across each domain are used to calculate domain scores. Domain scores are subsequently transformed to generate a 0–100 scale to facilitate comparison with WHOQOL-100. Transformed domain scores were used in the current study. Cronbach’s alphas for the physical, psychological, social, and environment domains were .90, .90, .76, .86, respectively.

### Procedure

Participants were invited to complete an anonymous online questionnaire via a combination of convenience and snowball sampling techniques. Social media platforms associated with ex-service organisations, veteran networking organisations, and researcher’s networks were used to disseminate the URL to the online questionnaire. No incentive or remuneration was offered. Participant parameters were guided by extant definitions of contemporary Australian Defence Force veterans [11, 72, 73]. Eligibility screening questions are common practice, in particular in online research, and provide ethical guidance for recruitment [74]. Governed by the aforementioned definitions, Australian Defence Force veterans who were actively serving or had not served in a full-time capacity were excluded. Also excluded were veterans who had discharged prior to 2000. Following Romaniuk [75], participants who were hospitalised for a psychological condition were excluded to minimise the risk of harm and meet low risk ethics requirements. To support comparison and generalisability of findings in an Australian and cross-cultural context, self-report demographic, Australian Defence Force experience, and transition experience questions were aligned to pertinent research at the time [2, 5–7, 11, 12, 16, 62, 63, 75, 76].

## Results

### Data preparation

Data were screened using SPSS 25.0. The total sample included 358 cases and there were no missing values. Three multivariate outliers (Mahalanobis distance > 22.46) were identified; however, cases were retained as Cook’s leverage statistics were within acceptable limits (range from < .001 to .062) [77]. Tolerance values (> .2) and variance inflation factor (VIF) estimates (< 10 and average VIF <2) were within acceptable limits, indicating multicollinearity was not a concern [77]. Data associated with the SCS-R and WHOQOL-Bref domains were approximately normally distributed. The maximum skewness and kurtosis values for WIS items (skew = -1.11; kurtosis = 1.16) and K10 scores (skew = .509; kurtosis = -.404) indicated data were not substantially non-normal and that the maximum likelihood method of estimation with bootstrapping was appropriate for analyses [78].

### Analysis plan

Analyses were conducted in two phases using AMOS 25.0. First, the 31-item WIS (S1 Table) was examined using Confirmatory Factor Analysis (CFA) to assess model fit and suitability for path analysis. Following the CFA, exploratory path analysis was conducted using the re-specified WIS. As the sample size was large and data were not substantially non-normal, the maximum likelihood method of estimation with bootstrapping was employed. Several fit indices were used to assess the goodness-of-fit of the measurement and structural models. The Root Mean Square Error of Approximation (RMSEA) ≤ .06 statistic was employed as an index of absolute fit, and the Comparative Fit Index (CFI) and Tucker-Lewis Index (TLI) values ≥ .95 were used as incremental fit indices [78, 79]. The chi-square test is an index of exact-fit and is sensitive to sample size, non-normality, and discrepancies [78]. Given the exploratory nature of this study, the chi-square statistic is reported for model comparison only. Areas of model misspecification were identity by modification indices, low standardized regression weights or factor loadings, and high correlation residuals [78]. Model re-specification was guided by theory, past research, and model fit.

### Confirmatory factor analysis

A single factor measurement model incorporating the 31 WIS items (refer to S1 Table for items) was specified and tested to rule out unidimensionality. As detailed in Table 2, the single factor model was a poor fit to the data. Thus, the 31-item seven-factor WIS was specified and tested with the factors permitted to covary. The fit statistics revealed the 31-item seven-factor model failed to reach an acceptable fit (see Table 2) and the modification indices revealed numerous items cross-loaded onto multiple factors and shared residual variance with items on non-specified factors. As model discrepancies are collapsed into an overall fit, misspecifications within factors were examined to refine the measurement model [78]. Factors with four or more items (private, public, interdependence, and centrality) were specified and tested as one-factor congeneric models [80]. The aim was to re-specify each of the congeneric models to improve the conceptual clarity of each factor by removing poor fitting items. This was achieved by removing items with low loadings (< .6). Items with high residual correlations were also considered as candidates for removal. The one-factor congeneric models were trimmed until the model fit indices indicated a good fit or until only three items remained. After trimming the one-factor congeneric models, the fit of the seven-factor model was re-evaluated. Specifically, items identified to have high residual correlation with non-specified factors were deleted to preserve the clarity of each of the factors.

**Table 2:** Summary of fit statistics for tested Warrior Identity Scale models.

					RMSEA 90% CI	
Model	χ^2^ (*df*)	CFI	TLI	RMSEA	Lower	Upper
Single factor	3635.432* (434)	.423	.382	.144	.139	.148
31-item seven-factor	1162.042* (413)	.865	.848	.071	.066	.076
24-item seven-factor	663.989* (231)	.895	.874	.072	.066	.079
21-item six-factor	369.529* (174)	.945	.934	.056	.048	.064
20-item six-factor (WIS-6)	298.124* (155)	.959	.949	.051	.042	.060

CFI = Comparative Fit Index. TLI = Tucker-Lewis = Index. RMSEA = Root Mean Square Error of Approximation. CI = Confidence Interval.

*p < .001.

Two items on the private factor, “I often regret my military service” and “I am ashamed of my military service”, were trimmed due to displaying high residual variances. Both items were also considered redundant with “I am proud to have served in the military”. Consistent with Lancaster et al. [18], “I am happy that I am a veteran” was additionally trimmed from the private factor as was considered to overlap with “I feel good about my military service”. Four items were trimmed from the interdependence factor due to low factor loadings (See S1 Table for items removed). Although congeneric testing revealed two items on the centrality factor displayed low loadings, they were retained and re-examined in the context of the full measurement model due to aim of maintaining at least three items per factor.

**Fig 1:**
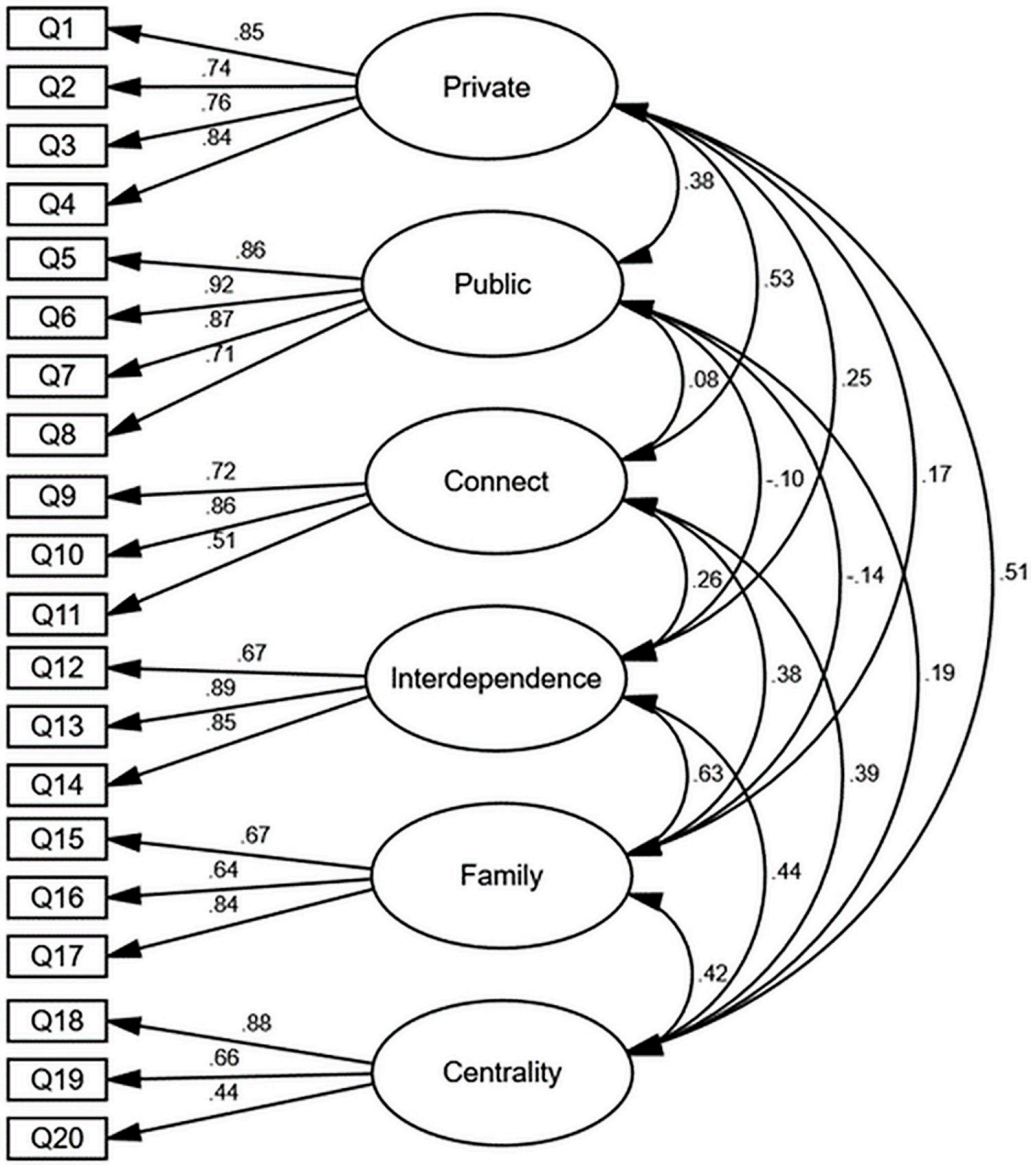
Revised 20-item six-factor Warrior Identity Scale (WIS-6) with standardised parameter estimates.

The revised 24-item seven-factor model was specified and tested. Items were permitted to only load onto their designated factor. Also, the factors were permitted to covary, although the item residuals were not. The 24-item seven-factor model displayed an improved but poor overall fit to the data (see Table 2). Examination of model revealed all three items on the skills factor demonstrated low factor loadings and several high residual correlations, indicating the items were not reflecting a unitary construct. Further inspection revealed multiple cross-loadings with non-specified factors. Subsequently, the skills factor was eliminated. As detailed in Table 2 the fit indices for the revised 21-item six-factor structure indicated a marked improvement, although the fit statistics remained suboptimal. Re-examination of the centrality factor revealed the item “Overall, having served in the military has very little to do with how I feel about myself” displayed the lowest factor loading and the highest residual correlations with several other items and was, therefore, removed. As detailed in Table 2, the final re-specified 20-item six-factor WIS structure (WIS-6) demonstrated a sound theoretical and good statistical fit to the data. Fig 1 provides a graphical representation of the re-specified WIS-6 with standardised parameter estimates (error terms are not depicted). Subscales were calculated, and Cronbach’s alpha for the private, public, connect, interdependence, family, and centrality subscales were .87, .90, .72, .84, .74, and .70, respectively.

### Path analysis

Prior to path analysis, interrelationships between variables of interest were examined using zero order Pearson product-moment correlations. Point biserial correlation was used for gender correlations (*N* = 357). Table 3 displays the correlations, means, and standard deviations of the variables of interest. A general trend indicated aspects of military identity increased with age and number of years served. However, age, number of years served in the military and gender did not significantly correlate with social connectedness or the outcome measures. Therefore age, years served and gender were not included as covariates. The conditional effects of social connectedness were tested by employing a nested model approach to assess the invariance of the relationships between each of the WIS-6 subscales and measures of wellbeing. To facilitate this, low and high social connectedness groups were determined by a median split on social connectedness measure. Equality constraints were imposed on the regression weights for the respective models to assess the stability of relationships between the WIS-6 subscales and wellbeing. A decrease in model fit indicates models are dissimilar. There was no significant change in the model fit when the regression paths were constrained to equality for the Kessler Psychological Distress, Δχ^2^ (6) = 10.516, *p* = .105; WHOQOL-Bref Physical Δχ^2^ (6) = 3.187, *p* = .875; WHOQOL-Bref Psychological Δχ^2^ (6) = 4.144, *p* = .657; WHOQOL-Bref Social; Δχ^2^ (6) = 5.922, *p* = .432; or the WHOQOL-Bref Environment Δχ^2^ (6) = 2.487, *p* = .870 models. Therefore, there was no support for a moderation effect of social connectedness.

**Table 3:** Correlations between 20-item six-factor Warrior Identity Scale and outcome variables, means, and standard deviations.

Scale/Measure	1	2	3	4	5	6	7	8	9	10	11	12	13	*M*	*SD*
1. Pri	-													13.63	2.53
2. Pub	.34**	-												10.07	2.65
3. Con	.45**	.076	-											9.65	1.94
4. Inter	.20**	-.14**	.21**	-										8.72	2.14
5. Fam	.17**	-.11*	.32**	.50**	-									8.54	2.18
6. Cen	.31**	.07	.33**	.40**	.38**	-								8.08	1.91
7. Phy^a^	.33**	.28**	.14**	-.29**	-.33**	-.11*	-							55.65	23.65
8. Psy^a^	.41**	.28**	.17**	-.28**	-.33**	-.11*	.77**	-						50.63	23.15
9. Soc^a^	.25**	.20**	.12*	-.23**	-.26**	-.13*	.53**	.70**	-					52.19	24.65
10. Env^a^	.32**	.32**	.15**	-.32**	-.37**	-.10*	.65**	.73**	.59**	-				61.44	19.24
11. K10	-.33**	-.26**	-.13*	.30**	.34**	.13*	-.75**	-.83**	-.61**	-.64**	-			23.92	9.46
12. SCS-R	.32**	.35**	.19**	-.33**	-.31**	-.16**	.62**	.76**	.64**	.69**	-.71**	-		69.24	21.64
13. Age	.26**	.14**	.20**	.10	.03	.20**	-.06	.06	-.01	.09	-.06	.02	-	42.05	9.49
14. Years	.23**	.05	.20**	.09	.00	.15**	-.06	.03	-.02	.10	-.01	-.01	.76**	14.45	8.64
15. Gender^b^	.06	-.01	.13*	.12*	.10	.06	-.00	.03	.01	-.01	.01	-.06	-	-	-

WIS-6 = Revised 20-item six-factor Warrior Identity Scale Pri = WIS-6 Private. Pub = WIS-6 Public. Con = WIS-6 Connect. Inter = WIS-6 Interdependence. Fam = WIS-6 Family. Cen = WIS-6 Centrality. Phy = WHOQOL-Bref Physical. Psy = WHOQOL-Bref Psychological. Soc = WHOQOL-Bref Social. Env = WHOQOL-Bref Environment. K10 = Kessler Psychological Distress Scale. SCS-R = Social Connectedness Scale–Revised. Years = Number of years served in the military.

^a^ Transformed to 0–100 scale.

^b^ Did not include unspecified gender.

* p < .05.

** *p* < .01.

The path models were subsequently specified and tested to explore the potential mediating effect of social connectedness on the relationship between military identity and wellbeing. Specifically, each of the WIS-6 subscales were modelled to exert their effect on the outcome measures via social connectedness. The analysis revealed significant paths existed between each of the WIS-6 subscales and social connectedness. As the modification indices indicated the model fit would be improved by specifying paths between the private and family subscales and all wellbeing measures with the exception of WHOQOL-Bref social domain model, models were re-specified with these paths. The additional direct paths failed to reach statistical significance in the WHOQOL-Bref social domain model (*p* > .05).

**Fig 2:**
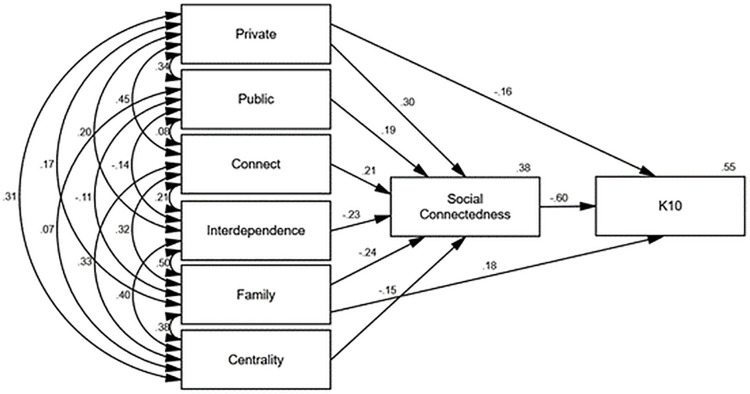
Final path model with standardised parameter estimates Kessler psychological distress scale (K10).

**Fig 3:**
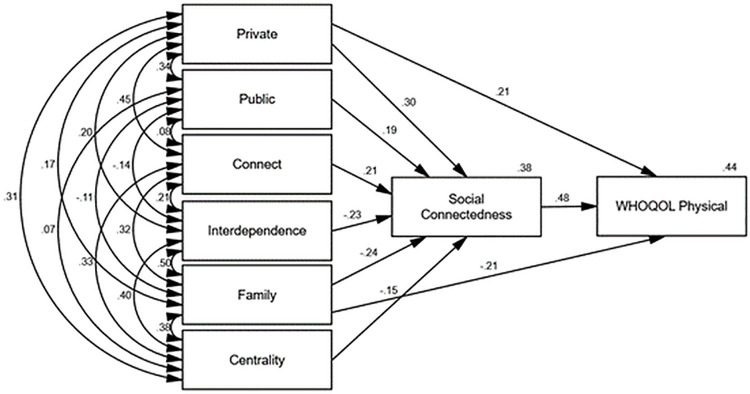
Final path model with standardised parameter estimates WHOQOL-Bref physical domain (transformed).

**Fig 4:**
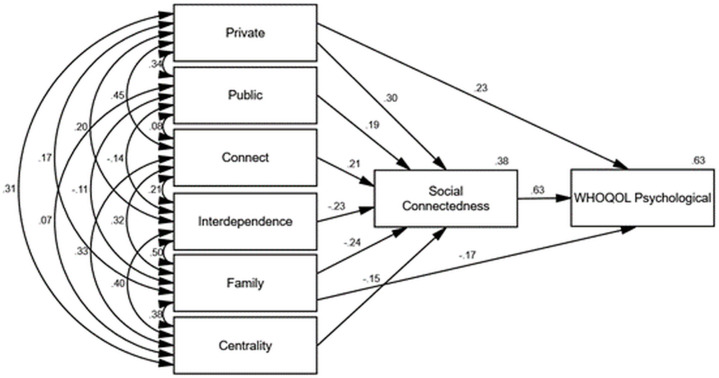
Final path model with standardised parameter estimates WHOQOL-Bref psychological domain (transformed).

**Fig 5:**
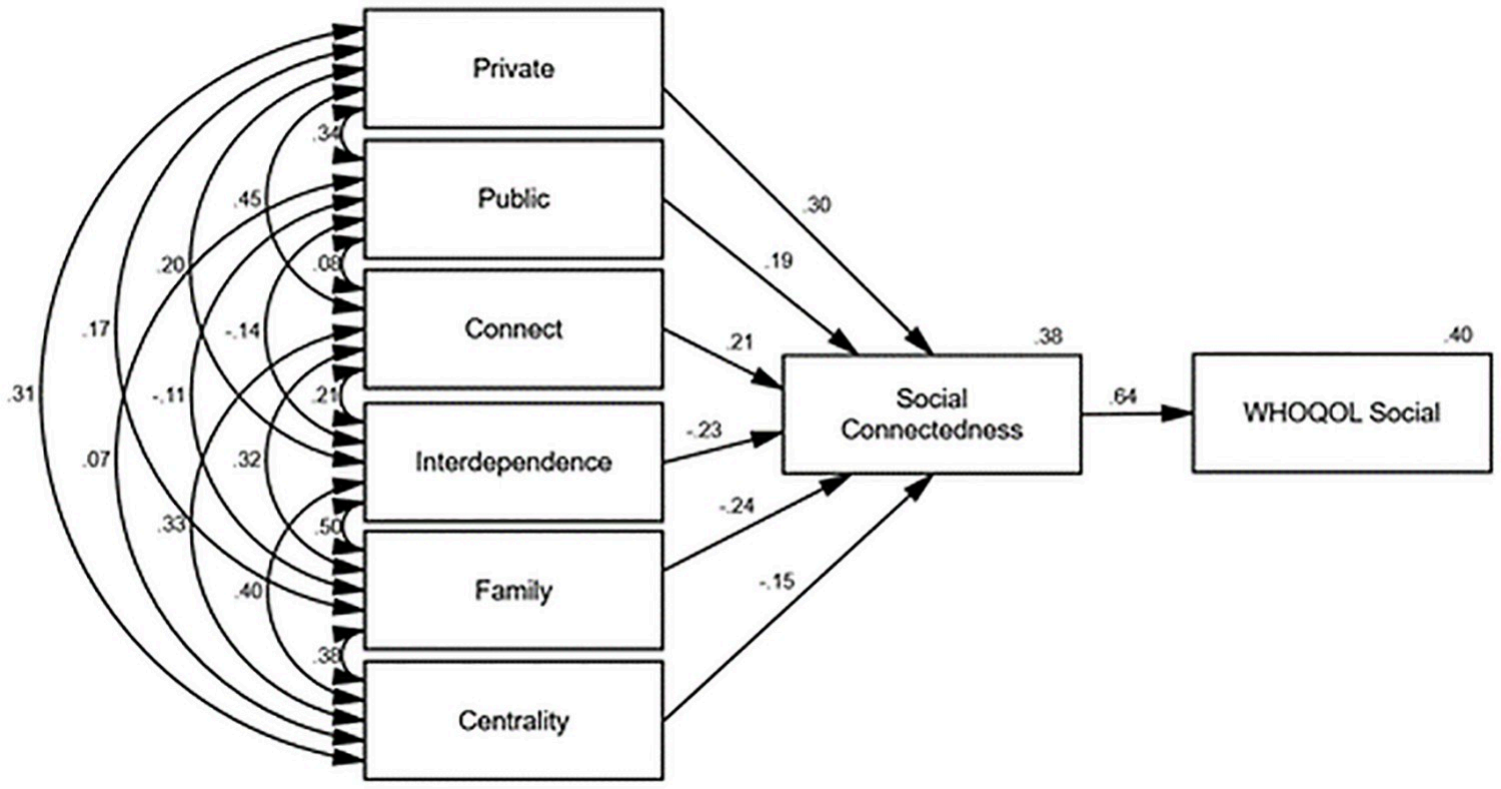
Final path model with standardised parameter estimates WHOQOL-Bref social domain (transformed).

**Fig 6:**
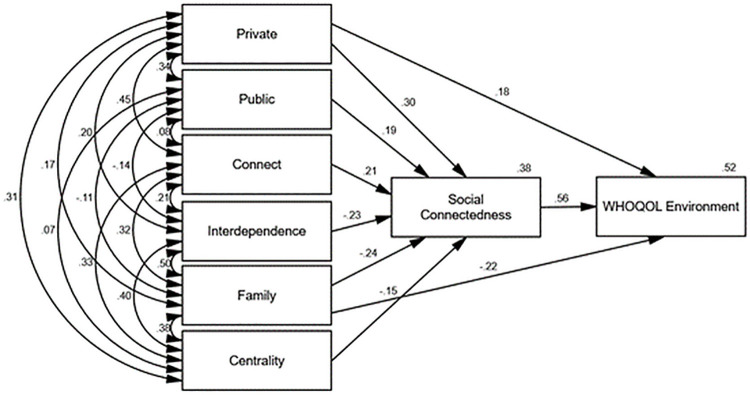
Final path model with standardised parameter estimates WHOQOL-Bref environment domain (transformed).

Figs 2–6 depict the final path models and standardised parameter estimates. All paths were significant at *p* < .05, and fit indices demonstrated all models were a good fit to the data (see Table 4). As depicted in Figs 2–6, social connectedness positively related to the WHOQOL-Bref domain scores and negatively related to K10 scores. Military identity accounted for 38% of the variance in social connectedness. Private, public, and connect subscales positively related to social connectedness, whereas interdependence, family, and the centrality subscales negatively related to social connectedness. With the exception of the WHOQOL-Bref social domain, which was fully mediated, the private and family subscales exerted additional direct effects on wellbeing measures. Social connectedness fully mediated the relationship between military identity and social wellbeing, explaining 40% of the variance in WHOQOL-Bref social domain scores. Whereas the private and family subscales in conjunction with social connectedness explained 55% of the variance in K10 scores, 44% of the variance in WHOQOL-Bref physical scores, 63% of the variance in WHOQOL-Bref psychological scores, and 52% of the variance in WHOQOL-Bref environment scores.

**Table 4:** Summary of fit statistics for path analysis models.

					RMSEA 90% CI	
Model	χ^2^ (*df*)	CFI	TLI	RMSEA	Lower	Upper
K10	2.968 (4)	1.00	1.009	.000	.000	.070
Physical^a^	5.697 (4)*	.999	.984	.034	.000	.093
Psychological^a^	4.565 (4)	.999	.996	.020	.000	.085
Social^a^	6.928 (6)	.999	.994	.021	.000	.074
Environment^a^	8.496 (4)	.995	.962	.056	.000	.109

CFI = Comparative Fit Index. TLI = Tucker-Lewis = Index. RMSEA = Root Mean Square Error of Approximation. CI = Confidence Interval. K10 = Kessler Psychological Distress Scale.

^a^ WHOQOL-Bref domain scores transformed to a 0–100 scale.

* p < .05.

## Discussion

The current study quantitatively explored the role of social connectedness in the relationship between military identity and subjective wellbeing among contemporary ex-serving Australian Defence Force veterans. Prior to examining potential moderating and mediating effects, the factor structure of the WIS was examined. Consistent with past research, CFA revealed a multidimensional construct of military identity [17, 18, 50, 67]. Subsequent re-specification of the WIS revealed a statistically and theoretically adequate 20-item six-factor measurement model suitable for further path analysis in an Australian Defence Force context. Similar to Kreminski et al. [16], no support was found for a moderation effect of social connectedness. Subsequently, the WIS-6 subscales were modelled to exert their effect on wellbeing via social connectedness. Results indicated military identity indirectly influenced wellbeing via social connectedness and that two aspects of military identity also exerted a direct influence on wellbeing. In line with military transition theory [30] and the social identity model of adjustment to identity change [22], the results indicate military identity and social connectedness should be examined conjointly to understand the interplay and relative importance of the risk and protective effects of these constructs and their influence on wellbeing among contemporary ex-serving veterans.

The associations between private, public, and the WIS interdependence subscales with social connectedness in the current study were consistent with the findings of Kintzle [19]. Collectively, military identity accounted for 38% of the variance in social connectedness. Consistent with past research, higher social connectedness related to higher quality of life and lower psychological distress [15, 19, 38]. The findings suggest the way ex-serving veterans identify with the military self is integral to shaping a sense of social connectedness, which in turn influences wellbeing. Specifically, high private and public regard for the military and not feeling like an outsider related to higher social connectedness. Conversely, interdependence with other veterans, viewing the military as family, and a centrality of military identity inhibited social connectedness.

In addition to the indirect influence of military identity on wellbeing via social connectedness, the private and family subscales also exerted direct influence on physical, psychological, and environment wellbeing measures. Consistent with previous research [17–19], this finding indicates a low private regard for the military and viewing the military as a family may be particularly problematic among ex-serving veterans as a result of the compounding detrimental effects to both social connectedness and wellbeing. Conversely, a high private regard and less reliance on the military and other veterans for social connection may be highly protective. Furthermore, the direct relationships between wellbeing and private and family identity suggest social connectedness does not exclusively explain the relationship between military identity and wellbeing.

The direction of the relationships between the aspects of military identity and measures of wellbeing and social connectedness explored in the current study are consistent with previous research investigating with the WIS in clinical and non-clinical ex-serving US veteran samples [17, 18]. However, the current study extends previous research by examining the pathways between military identity, social connectedness, and subjective wellbeing among contemporary ex-serving Australian Defence Force veterans. The current study demonstrates how aspects of military identity can support or inhibit a sense of social connectedness which, in turn, influences subjective wellbeing. In addition, the findings demonstrate that a private regard for the military and viewing the military as a family operate over and above social connectedness to influence wellbeing.

The current study also expands on earlier work by Kreminski et al. [16] who found protective effects of a perceived connectedness to former military networks and negative effects of a centrality of military identity on wellbeing in an Australian military sample. Additionally, the findings expand on earlier qualitative and mixed-methods research by quantitatively demonstrating how an ensuing military identity in conjunction with a disconnect from civilian society may interrelate to influence wellbeing [5, 30, 42, 45–47, 53–58, 60]. Furthermore, the findings contribute to the body of research evidencing the multidimensional nature of military identity. Importantly, the current study highlights the protective and inhibiting effects of military identity to both social connectedness and wellbeing that may otherwise be obfuscated by a unidimensional focus on military identity [17, 18, 50, 51, 67].

The current findings indicate interventions that consider the different aspects of military identity while promoting broader social connections maybe particularly effective. That is, by fostering social connections of ex-serving members with veteran groups and other social groups may help in the adjustment to the non-centrality of military life, as well as improve mental health outcomes. Importantly, a sense of social connectedness is not fixed, and can be established where individuals experience trusting relationships, are not rejected, and feel a sense of collective identity [31]. As such, peer-support and group-based behavioural activation programs provide striking examples of how military identity and social connectedness may interrelate to influence wellbeing [81–90]. For instance, veteran-centred therapeutic horseback riding [88], surfing [89], and group exercise programs [86] have demonstrated an ability to reduce PTSD symptoms and improved social connectedness among ex-serving US veterans. Improved social connectedness and wellbeing has also been reported among contemporary ex-serving Australian veterans who participated in a peer outdoor support therapy program [85]. Research is also emerging in support of the improved social connectedness among ex-serving veterans attending programs without a clinical focus such as social networking groups [85, 87].

### Limitations and future research

Due to time constraints and the unique nature of the target population, a range of ex-service organisations and social networking groups assisted with participant recruitment. As such, it is possible the sample may reflect ex-serving Australian Defence Force veterans who have a higher degree of social connectedness compared to the population of interest. Additionally, the cross-sectional design and sampling method may have introduced sampling bias, as the student researcher was a serving member of the Australian Army at the time of the study. Furthermore, the online anonymous survey design may have introduced self-report bias. These factors limit conclusions relating to the suitability of the WIS and path analysis findings to other populations. However, the large sample size and comprehensive sample profile provides some degree of confidence in the representativeness of the sample and generalizability of the findings.

The design of the current study considered the combined effects of the WIS-6 factors on wellbeing via social connectedness. Given the differential influences of the aspects of military identity on both social connectedness and wellbeing, it is likely multiple profile combinations may exert different effects on wellbeing. Additionally, the current study explored pathways using a non-clinical sample of participants. As such, future research could examine problematic and protective profiles via cluster analyses in both clinical and non-clinical populations. This approach may further inform veteran-centric intervention programs aimed at improving the social and individual wellbeing of contemporary ex-serving veterans.

Despite the limitations, the current study provided support to the multidimensional construct of military identity, and the suitability of the WIS for further analysis in an Australian Defence Force context. The findings suggest wellbeing following transition from military to civilian life is influenced by the complex interrelationships between aspects of military identity and a sense of social connectedness. From a practical perspective, the results suggest nurturing the protective aspects of military identity and addressing problematic aspects of military identity may support a sense of social connectedness and wellbeing among ex-serving veterans. Furthermore, the significance of a sense of social connectedness to wellbeing indicates interventions targeting improved social connectedness among veteran populations may not only directly improve wellbeing but may also influence the way veterans identify with the military self and connect to the social world following transition from military to civilian life.

## Supporting information

S1 Table 31-item Warrior Identity Scale (March 29, 2019, email from Lancaster, S.L., Assoc Professor and Chair, Department of Psychology, Bethel University MN: Steven-lancaster@bethel.edu). (PDF)
