# Supplementary material for: Transition from military to civilian: Identity, social connectedness, and veteran wellbeing
Source: PLoS One. 2021 Dec 22;16(12):e0261634. doi: 10.1371/journal.pone.0261634 (PMC8694481; doi:10.1371/journal.pone.0261634)
Supplement: S1 Table — (PDF) [file pone.0261634.s001.pdf]

S1 Table. 31-Item Warrior Identity Scale

Scale/Item
Private
1. # I am happy that I am a veteran.
2. I feel good about my military service (Item 1 WIS-6).
3. I am proud of the things that veterans have accomplished (Item 2 WIS-6).
4. I believe that I have many strengths due to my military service (Item 3 WIS-6).
5. # I often regret my military service.
6. I am proud to have served in the military (Item 4 WIS-6).
7. # I am ashamed of my military service.
Interdependent
8. Only other veterans can truly understand me (Item 12 WIS-6).
9. When I meet other veterans I can trust them more quickly than other people (Item 13 WIS-6).
10. I become friends with other veterans more quickly than with non-veterans (Item 14 WIS-6).
11. # My fate and future are bound up with that of veterans.
12. # Regarding other veterans, it is accurate to say, "United we stand, divided we fall."
13. # The most important things that have happened in my life involve my military service.
14. # When I talk about the military, I usually say 'we' rather than 'they.'
Connect
15. During my time within my unit in the military I always felt like an outsider (Item 9 WIS-6).
16. I never felt emotionally connected to my military unit (Item 10 WIS-6).
17. Throughout my time in the military I resisted believing in military rituals and norms (Item 11 WIS-6).
Family
18. I miss my military friends (Item 15 WIS-6).
19. I wish I could go back into the military (Item 16 WIS-6).
20. By leaving the military I lost a family (Item 17 WIS-6).
Centrality
21. # Overall, having served in the military has very little to do with how I feel about myself.
22. In general, being a veteran is an important part of my self-image (Item 18 WIS-6).
23. Being a veteran is unimportant to my sense of what kind of person I am (Item 19 WIS-6).
24. Being a veteran is not a major factor in my social relationships (Item 20 WIS-6).
Public Regard
25. Overall, veterans are highly thought of (Item 5 WIS-6).
26. In general, others respect veterans and members of the military (Item 6 WIS-6). .
27. In general, other groups view veterans in a positive manner (Item 7 WIS-6).
28. Society views veterans as an asset (Item 8 WIS-6).
Skills
29. # I appreciate the skills I learned in the military.
30. # The work I do at home has more meaning for me than the work I did for the military
31. # I miss the job related aspects of my time in the military.

Note: Items (5, 7, 15-17, 21, 23, 24, and 30) are Reverse scored; # Items/factor deleted from WIS-6
